# Evaluation of a fully automated bioinformatics tool to predict antibiotic resistance from MRSA genomes

**DOI:** 10.1093/jac/dkz570

**Published:** 2020-02-05

**Authors:** Narender Kumar, Kathy E Raven, Beth Blane, Danielle Leek, Nicholas M Brown, Eugene Bragin, Paul A Rhodes, Julian Parkhill, Sharon J Peacock

**Affiliations:** 1 Department of Medicine, University of Cambridge, Box 157, Addenbrooke’s Hospital, Hills Road, Cambridge CB2 0QQ, UK; 2 Clinical Microbiology and Public Health Laboratory, Public Health England, Cambridge CB2 0QQ, UK; 3 Next Gen Diagnostics, LLC (NGD), Mountain View, CA, USA and Wellcome Genome Campus, Hinxton, Cambridge CB10 1SA, UK; 4 Department of Veterinary Medicine, University of Cambridge, Cambridge, UK

## Abstract

**Objectives:**

The genetic prediction of phenotypic antibiotic resistance based on analysis of WGS data is becoming increasingly feasible, but a major barrier to its introduction into routine use is the lack of fully automated interpretation tools. Here, we report the findings of a large evaluation of the Next Gen Diagnostics (NGD) automated bioinformatics analysis tool to predict the phenotypic resistance of MRSA.

**Methods:**

MRSA-positive patients were identified in a clinical microbiology laboratory in England between January and November 2018. One MRSA isolate per patient together with all blood culture isolates (total *n *=* *778) were sequenced on the Illumina MiniSeq instrument in batches of 21 clinical MRSA isolates and three controls.

**Results:**

The NGD system activated post-sequencing and processed the sequences to determine susceptible/resistant predictions for 11 antibiotics, taking around 11 minutes to analyse 24 isolates sequenced on a single sequencing run. NGD results were compared with phenotypic susceptibility testing performed by the clinical laboratory using the disc diffusion method and EUCAST breakpoints. Following retesting of discrepant results, concordance between phenotypic results and NGD genetic predictions was 99.69%. Further investigation of 22 isolate genomes associated with persistent discrepancies revealed a range of reasons in 12 cases, but no cause could be found for the remainder. Genetic predictions generated by the NGD tool were compared with predictions generated by an independent research-based informatics approach, which demonstrated an overall concordance between the two methods of 99.97%.

**Conclusions:**

We conclude that the NGD system provides rapid and accurate prediction of the antibiotic susceptibility of MRSA.

## Introduction

There is growing evidence for the potential of pathogen sequencing to transform infection control practice and outbreak investigation.^1–6^ As a result, sequencing technologies are becoming increasingly employed in diagnostic and public health microbiology laboratories for surveillance, outbreak investigation and transmission tracking of hospital and foodborne-associated outbreaks and emerging pathogens. The reuse of such sequence data to also detect genetic mutations and acquired genes associated with phenotypic antibiotic resistance could provide a rich source of surveillance information at little or no additional cost. Accuracy of the genetic prediction of phenotypic resistance depends on access to a comprehensive reference database but, if made available, sequence data could be used to support clinical care and provide an additional mechanism for the quality control (QC) of routine phenotypic testing.

As the cost and turnaround time of sequencing technologies fall and the databases necessary for genetic prediction expand, genome sequencing will also become adopted as the primary method to detect genetic determinants of resistance. This is already the case for *Mycobacterium tuberculosis*, with sequencing having entered into routine practice for prediction of resistance and outbreak investigation in several countries.^7^ This change in methodology is readily justified as susceptibility testing is laborious and requires considerable expertise, whilst sequencing is cost-effective and potentially more rapid.^8^ The case for using sequencing as a primary method to detect resistance genes in rapidly growing bacteria includes the detection of MDR pathogens that are currently evaluated using directed molecular methods such as PCR.^9^^,^^10^ Furthermore, genome sequencing has the advantage of providing information on the entire genetic repertoire, compared with amplification methods that target a small number of specific genes.

Although rapid progress has been made to develop pathogen sequencing for use in routine diagnostic and public health microbiology, a critical rate-limiting step is the lack of fully automated interpretation tools for use by those with limited informatics training. Here, we report the findings of a large prospective evaluation of the Next Gen Diagnostics (NGD) automated bioinformatics tool to predict the phenotypic resistance of MRSA.

## Materials and methods

### Ethics approval, study setting, patients and sample identification

The study was conducted under ethics approval from the National Research Ethics Service (ref: 11/EE/0499) and the Cambridge University Hospitals NHS Foundation Trust Research and Development Department (ref: A092428). The study setting was the Clinical Microbiology and Public Health Laboratory at the Cambridge University Hospitals NHS Foundation Trust (CUH) in the UK. MRSA-positive patients with samples submitted between 24 January 2018 and 1 November 2018 were identified using the hospital IT system [EPIC EMR (Hyperspace^®^ 2014; Epic Systems Corporation)]. From 24 January to 2 April 2018, isolates were retrieved from the frozen archive in the clinical laboratory. From 3 April to 1 November 2018, putative or confirmed MRSA-positive culture plates were retrieved prospectively by the research team. The species was confirmed as *Staphylococcus aureus* using the Staph Latex kit (Pro-Lab Diagnostics). Freezer archive samples were plated onto Columbia Blood Agar (CBA) and incubated overnight at 37°C. For samples obtained prospectively, a single colony, where possible, was selected from the clinical plate (several colonies or a 1 μL loopful was taken where colonies were smaller than 2 mm or growth was confluent), plated onto CBA and incubated overnight at 37°C. A 10 μL loopful was then stored at −80°C in Microbank vials (Pro-Lab Diagnostics). The first available isolate from each patient was selected, together with all blood culture isolates. Laboratory data were collected on type of specimen, sampling date and location (hospital or GP). Patients and samples were recoded with an anonymous study number prior to further evaluation. We identified 786 MRSA isolates (from 782 patients) that were cultured from samples submitted to the laboratory during the study period, but 8 isolates (8 patients) were subsequently excluded from the study because of laboratory error (*n *=* *3) or contamination (*n *=* *5). The 778 study isolates are listed in Table S1 (available as Supplementary data at *JAC* Online).

Susceptibility testing was performed by the clinical laboratory using the disc diffusion method and EUCAST breakpoints (http://www.eucast.org/fileadmin/src/media/PDFs/EUCAST_files/Disk_test_documents/2019_manuals/Manual_v_7.0_EUCAST_Disk_Test_2019.pdf), with a panel of 6, 9 or 12 antibiotics based on standard laboratory protocols. The 12-antibiotic panel (cefoxitin, chloramphenicol, ciprofloxacin, erythromycin, fusidic acid, gentamicin, linezolid, mupirocin, neomycin, rifampicin, trimethoprim/sulfamethoxazole and tetracycline) was used to test isolates from blood cultures and/or patients either not known to be MRSA-positive or without a 12-antibiotic panel in the prior year. The 9-antibiotic panel (cefoxitin, chloramphenicol, ciprofloxacin, gentamicin, linezolid, mupirocin, neomycin, trimethoprim/sulfamethoxazole and trimethoprim) was used to test MRSA-positive urine cultures. The 6-antibiotic panel (cefoxitin, erythromycin, tetracycline, rifampicin, fusidic acid and gentamicin) was used to test isolates from non-invasive infections from GP surgeries. A minority of MRSA cultured from multisite screens of known MRSA patients were confirmed as cefoxitin resistant but had no further susceptibility testing performed.

Repeat disc diffusion testing, Etests and broth microdilutions for the discrepant isolates were performed in the research laboratory from frozen stocks, which were plated onto CBA and incubated at 37°C for 24 h. A single colony was selected for susceptibility testing according to the EUCAST guidelines. For fusidic acid (for which there was no Etest available) repeat testing was performed using the broth microdilution method. For some isolates (see the Results section), we undertook a combination of repeat phenotypic testing and sequencing from a single colony. Frozen stocks were plated onto CBA and incubated at 37°C for 24 h, after which a single colony was plated onto CBA and incubated at 37°C for 24 h to create a single-colony purity plate. One colony was taken from this purity plate for sequencing and a second was used for disc testing.

### WGS and QC

Frozen stocks were plated onto CBA and incubated at 37°C for 24 h, then a single colony taken for DNA extraction. DNA extraction, library preparation and sequencing were performed as described previously.^11^ In brief, DNA was extracted using the QIAGEN DNA Mini Extraction Kit, sequencing libraries were made using the Illumina Nextera DNA Flex Kit and sequencing was performed on an Illumina MiniSeq with a run time of 13 h using the high-output 150 cycle MiniSeq cartridge and the Generate FASTQ workflow. Each run contained 21 clinical MRSA isolates and three controls [no template (water), positive control (MRSA MPROS0386) and negative control (*Escherichia* *coli* NCTC 12241)]. The study sequences are available in the European Nucleotide Archive (https://www.ebi.ac.uk/ena) under the accession numbers provided in Table S1. Controls were required to pass predefined quality metrics. The positive control was required to: have the highest match to *S. aureus* using Kraken; be assigned to ST22; have *mec* detected (>70% identity, >90% length); and have a minimum mean sequence depth of 20× and minimum 80% mapping coverage of the MRSA reference genome (HO 5096 0412). The negative control was required to: have the highest species match to *E. coli* using Kraken; not have *mec* detected; and have no *S. aureus* ST assigned. The ‘no template’ control was required to have less than 1% contamination from any bacterial DNA. In addition, each sequenced isolate was subjected to the following QC assessment: highest match to *S. aureus* using Kraken; *mec* detected; minimum sequence depth of 20× and minimum 80% coverage of the MRSA reference genome (HO 5096 0412).

### Sequence data analysis using standard bioinformatics pipelines

Bacterial species were determined using Kraken version 1 (https://ccb.jhu.edu/software/kraken/) and the miniKraken database (https://ccb.jhu.edu/software/kraken/dl/minikraken_20171019_8GB.tgz). STs were identified for MRSA using ARIBA version 2.12.1, as described at https://github.com/sanger-pathogens/ariba/wiki/MLST-calling-with-ARIBA. We used a database of genes and mutations described previously as conferring resistance in *S. aureus* (Table S2).^12^^,^^13^ Presence of genes and mutations was determined using ARIBA version 2.12.1 run using the default settings. The output was filtered using a custom script (10.6084/m9.figshare.11316665), with a gene classified as present if there was >90% identity match, >90% of the gene length was assembled and the coverage depth of the reference gene was less than 2 SD below the average genome coverage. If a gene did not pass these parameters then the isolate was classed as genotypically susceptible by our research pipeline.

### Sequence data analysis using an automated system

The NGD automated bioinformatics tool version 0.1.0 beta predicts antibiotic resistance/susceptibility for a total of 32 antimicrobial agents (Table S3) and provides details of the genes or genetic mutations used as the basis for resistance prediction. The system self-activated on upload of the MiniSeq output file and processed the 24 pairs of FASTQ files. The raw reads were trimmed with Trimmomatic version 0.36.4^14^ to remove low-quality bases from the ends of each read and filter out reads with low average base-pair quality. Genomes were flagged as passed or failed based on having 20× coverage over at least 80% of the mapping reference. Each MRSA genome was assessed for the presence of predefined genes and mutations that are known to confer resistance, utilizing a proprietary resistance database, which was compiled from available public literature and datasets, curated and validated internally. The database includes annotations for minimum identity, gene length and absolute/relative depth required for each individual gene and/or mutation. Resistance and susceptibility predictions were automatically generated. The speed of the tool was determined based on the time it took to generate the susceptible/resistant prediction report from the uploaded MiniSeq output file for a single run of 24 isolates in triplicate (11 min 11 s, 11 min 0 s and 10 min 53 s, respectively) and three independent sequence runs of 24 isolates (11 min 11 s, 9 min 54 s and 11 min 20 s, respectively). These resulted in a range of 9 min 54 s to 11 min 20 s, with an average of 10 min 52 s. Isolates were classified as ‘uncertain’ by the NGD analysis tool in the event that the sequence data did not pass internal QC metrics. These were defined as ‘inconclusive results’ and excluded from the primary analysis. This occurred for 19 out of a total of 7147 possible isolate–antibiotic combinations that could be evaluated (0.27%), the cause of which was determined as gene coverage and/or gene coverage depth below the required threshold (Table S4). These underwent repeat testing to determine the cause of the failure, the findings from which are described in the Results section.

## Results

We evaluated 778 isolates from 774 MRSA-positive individuals that were cultured from samples submitted to the laboratory between January and November 2018 from wards and clinics at three hospitals (*n *=* *639) and 65 GP surgeries (*n *=* *139). The majority of samples were multisite screens (swabs of nose, throat and groin, *n *=* *524), the remainder being diagnostic specimens including swabs (*n *=* *221), tissue (*n *=* *10), respiratory samples (*n *=* *9), blood cultures (*n *=* *7), urine (*n *=* *5) and other body fluids (*n *=* *2). *In silico* MLST prediction demonstrated that the 778 isolates belonged to 66 different STs, including 16 isolates with a novel ST. The most common STs were ST22 (47%), ST45 (10%) and ST59 (8%) (Table S1 and Figure S1).

The clinical laboratory undertook phenotypic susceptibility testing against a total of 13 antibiotics. Twelve antibiotics were tested for the majority of isolates (*n *=* *687, 88.3%), the remainder being tested against nine or six antibiotics (5 and 31 isolates, respectively) or cefoxitin alone (55 isolates; see methodology for rationale of variable testing). The number of isolates tested per antibiotic was as follows: cefoxitin (*n *=* *778), erythromycin (*n *=* *719), tetracycline (*n *=* *718), rifampicin (*n *=* *718), fusidic acid (*n *=* *718), gentamicin (*n *=* *723), chloramphenicol (*n *=* *692), mupirocin (*n *=* *692), linezolid (*n *=* *692), neomycin (*n *=* *692), trimethoprim/sulfamethoxazole (*n *=* *692), ciprofloxacin (*n *=* *692) and trimethoprim (*n *=* *5). Two antibiotics (neomycin and trimethoprim/sulfamethoxazole) were excluded from further analysis because the resistance determinants were not included in the NGD reference database and so the NGD tool could not predict resistance for these. The proportion of isolates with resistant, intermediate and susceptible phenotypes varied by drug (Figure S2). Antibiotics with the highest proportion of resistant isolates were ciprofloxacin (60%, 412/692), erythromycin (46%, 330/719) and fusidic acid (32%, 228/718). There were 7128 possible isolate–antibiotic combinations that passed the NGD tool QC and could be evaluated for the accuracy of phenotypic antibiotic susceptibility prediction. We excluded 99 results (1.4%) associated with intermediate resistance from further analysis because the NGD tool provides resistant or susceptible predictions.

**Figure 1.**
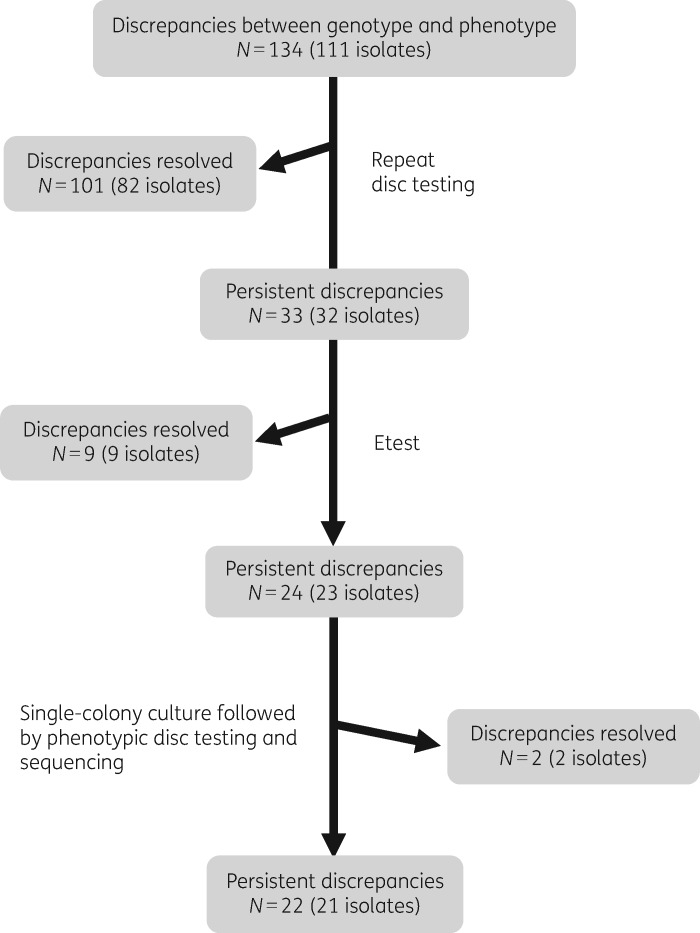
Algorithm used for retesting of discrepancies between results of phenotypic testing performed by the clinical laboratory and genetic prediction of susceptible/resistant status using the NGD tool.

A total of 7029 isolate–antibiotic combinations were used to evaluate the accuracy of the NGD tool. This demonstrated that NGD tool predictions were concordant with the clinical laboratory phenotype results in 6895/7029 (98.09%) instances. The 134 discordant pairs involved 10 antibiotics, the most common of which was ciprofloxacin (Figure S3). These were further evaluated by the retesting algorithm shown in Figure 1, which resolved the majority of discrepancies (112/134, 83.6%) (Table S5). Recalculation of the concordance between the actual and predicted phenotypic using the NGD tool gave a concordance of 99.69% (7007/7029), with a very major error rate of 0.72% and a major error rate of 0.15%. The final comparison metrics are shown in Table 1.

**Table 1.** Evaluation of the genetic predictions of antibiotic susceptible/resistant phenotype made by the NGD tool for 778 MRSA isolates after repeat testing for discrepancies

Drugs	True positives	True negatives	False positives	False negatives	Accuracy (%)	Sensitivity (%)	Specificity (%)	Very major errors (%)	Major errors (%)
Cefoxitin	778	0	0	0	100	100	NA	0	NA
Erythromycin	324	384	1	1	99.72	99.69	99.74	0.31	0.26
Tetracycline	101	609	0	0	100	100	100	0	0
Rifampicin	8	699	0	0	100	100	100	0	0
Fusidic acid	220	494	1	1	99.72	99.55	99.79	0.45	0.21
Gentamicin	53	668	0	0	100	100	100	0	0
Chloramphenicol	4	684	0	4	99.42	50	100	50	0
Mupirocin	31	571	6	0	99.01	100	98.96	0	1.04
Linezolid	0	692	0	0	100	NA	100	NA	0
Ciprofloxacin	398	289	0	5	99.28	98.76	100	1.24	0
Trimethoprim	0	0	0	3	0	NA	NA	NA	NA
Overall	1917	5090	8	14	99.69	99.27	99.84	0.72	0.15

Definitions: True positive, both phenotype and genotype are resistant; True negative, both phenotype and genotype are susceptible; False positive, phenotype is susceptible but genotype is resistant; False negative, phenotype is resistant but genotype is susceptible.

NA (not applicable) refers to cases where a particular value could not be calculated because of insufficient data (for example, no cases of resistance).

Investigation of the 22 isolate genomes associated with persistent discrepancies between phenotypic testing and predicted phenotype by NGD revealed an explanation in 12 cases (Table 2). Seven isolates with phenotypic susceptibility to mupirocin were predicted as resistant by the NGD tool based on the presence of an isoleucine t-RNA synthetase gene (*ileS-2* or *mupA*). However, all seven isolates had a frameshift mutation at the 93rd codon of *ileS-2* that would be predicted to cause functional inactivation of the *mupA* gene and a susceptible phenotype. Four isolates with phenotypic resistance to chloramphenicol carried *fexA*, which encodes chloramphenicol resistance^15^ but this gene was missing from the NGD database and the isolates were predicted as being susceptible. One isolate with phenotypic susceptibility to fusidic acid was predicted as resistant by the NGD tool based on a M453I mutation in *fusA*, but this mutation has not been described previously as conferring resistance.^16^ One isolate (HICF0659) with phenotypic resistance to trimethoprim was predicted as susceptible by the NGD tool, but the isolate carried *dfrA*, which confers resistance.^15^ Reasons for the remaining nine discrepancies could not be identified.

**Table 2.** Twenty-two discrepancies between the result from phenotypic testing and NGD tool predictions that were not resolved by repeat testing

Strain ID	Drug	Phenotype	NGD tool prediction	Gene detected
HICF0228	chloramphenicol	R	S	—
HICF0465	chloramphenicol	R	S	—
HICF0561	chloramphenicol	R	S	—
HICF0696	chloramphenicol	R	S	—
HICF0214	ciprofloxacin	R	S	—
HICF0413	ciprofloxacin	R	S	—
HICF0441	ciprofloxacin	R	S	—
HICF0525	ciprofloxacin	R	S	—
HICF0941	ciprofloxacin	R	S	—
HICF0441	erythromycin	R	S	—
HICF0065	fusidic acid	R	S	—
HICF0838	fusidic acid	S	R	*fusA* M453I
HICF0006	mupirocin	S	R	*ileS-2* ^a^
HICF0099	mupirocin	S	R	*ileS-2* ^a^
HICF0201	mupirocin	S	R	*ileS-2* ^a^
HICF0366	mupirocin	S	R	*ileS-2* ^a^
HICF0401	mupirocin	S	R	*ileS-2* ^a^
HICF0828	mupirocin	S	R	*ileS-2* ^a^
HICF0890	mupirocin	S	R	*ileS-2* ^a^
HICF0659	trimethoprim	R	S	—
HICF0372	trimethoprim	R	S	—
HICF0802	trimethoprim	R	S	—

S, susceptible; R, resistant; a dash indicates no detection of a known gene or genetic mutation associated with resistance.

a A frameshift mutation at the 93rd codon in these genes was detected that would be predicted to lead to gene inactivation, which would result in a susceptible phenotype.

Nineteen NGD tool prediction results (0.27%) were excluded prior to primary analysis because of failure to pass QC metrics and were assigned as inconclusive (see the Materials and methods section). These were re-evaluated by repeat phenotypic testing and sequencing from a single-colony purity plate (17 isolates). After retesting, 18 results were concordant between the phenotypic testing and the predicted phenotype, while one isolate (HICF0321) was phenotypically resistant to trimethoprim but no resistance gene was detected by the NGD tool.

Finally, we compared the performance of the NGD tool with a research pipeline in predicting the correct phenotypic susceptibility/resistance for the 7029 isolate–antibiotic combinations, using the dataset containing the 112 resolved discrepancies. The two pipelines were concordant for 99.97% (7027/7029) results. One isolate (HICF0659) with phenotypic resistance to trimethoprim was predicted to be susceptible by the NGD tool but the research pipeline detected *dfrA*, encoding trimethoprim resistance. One isolate with phenotypic resistance to fusidic acid was predicted as resistant by the NGD tool but susceptible by the research tool initially because although the *fusC* gene was detected in the research pipeline, it was below a QC cut-off metric. This discrepancy was resolved by repeat sequencing.

## Discussion

Here, we describe the outcome of a large evaluation of an automated bioinformatic system that enabled the rapid prediction of phenotypic antibiotic susceptibility for MRSA. Prediction was achieved with a high degree of accuracy, which is consistent with published studies on MRSA using research-based, non-automated informatics analyses.^12^^,^^17^ The advantage of the NGD tool was that it generated data from fully automated analyses for a complete run of 24 isolates/controls in the absence of bioinformatics expertise. Information was provided in a clinically relevant format (susceptible/resistant) together with details of the genes or genetic mutations used as the basis for resistance prediction. Processing time from DNA extraction to the generation of resistance predictions can be completed within 24 h, which brings the use of genomic technologies closer to clinical use for MRSA.

The final concordance between phenotypic susceptibility and predicted susceptibility/resistance was 99.69%, following retesting of 134 discordant pairs and resolution of discrepancies for 112 of these pairs. The very major error rate of 0.72% and major error rate of 0.15% falls well within the acceptable limits set by the FDA (<1.5% for very major errors and <3% for major errors). The NGD tool database did not contain two genes (*dfrA* and *fexA*) that confer resistance to trimethoprim and chloramphenicol, respectively. These have now been added to further increase the accuracy of prediction; this underlines the importance of an ongoing process of updating the supporting resistance database.

Our systematic study design was specifically used so that we could evaluate MRSA in a real-world clinical setting and avoid selection bias, whereby consecutive isolates were tested over 9 months from a single hospital. A limitation of this, however, is that the rate of resistance for some antibiotics (rifampicin, chloramphenicol, linezolid and trimethoprim) were very low in our setting, which impacts on the robustness of sensitivity and specificity calculations. This could be addressed by a future laboratory-based study in which collections are biased towards a higher proportion of resistant isolates. A study of *S. aureus* that also included methicillin-susceptible isolates (rather than only MRSA as in this study) would also confirm the accuracy of the tool for the prediction of cefoxitin resistance although the tool detects *mec* genes, which is an established approach for the prediction of resistance. The evaluation was limited to the analysis of Illumina paired-end data and we recognize that other sequencing technologies could become increasingly adopted in clinical laboratories over time.

Based on the output from each step of the retesting algorithm, most of the discrepancies identified during the initial assessment of concordance between disc diffusion test results and the NGD tool prediction were due to an erroneous disc diffusion test result, with 101/134 discrepancies (in 82 isolates) corrected after repeat disc diffusion testing. The direction of change in result after repeating the same assay was most commonly from resistant to susceptible (*n *=* *74), which is consistent with inaccuracies in clinical laboratory testing relating to the inoculum. The scale of the error rate (based on 101 errors out of 7029 individual results, 1.4%) is relatively low, but the findings from our study confirm that genome prediction can be more accurate overall than phenotypic testing. Furthermore, detection of laboratory errors based on the results of genetic predictions represents an additional mechanism for audit and quality improvement. One possibility that we took account of in our retesting algorithm was that picking a different colony for phenotypic testing and sequencing could lead to discrepant results if samples contain more than one strain, each of which had differing patterns of resistance. However, testing of persistently discrepant pairs from the same colony indicated that a change in result following this variation in methodology was the exception, suggesting that this does not represent a problem in practice.

An investigation of 22 persistently discrepant pairs after retesting provided important insights into mechanisms for this. Seven isolates contained *mupA*, encoding for mupirocin resistance, but analysis detected a frameshift mutation in this gene, which is likely to explain the susceptible phenotype. Incorporation of such mutations into the database will further improve the prediction accuracy, once the association between the mutation and susceptibility has been verified by relevant experimental testing. We also identified nine isolates that were apparently resistant but had no identifiable genetic cause based on current knowledge, which provides new avenues for the exploration of the basis of resistance. We excluded a small proportion of isolates with putative phenotypic intermediate resistance or issues relating to an intermediate resistance phenotype, including 10 isolates with mutations in the *ileS-1* gene that we knew would be predicted to confer full resistance by the NGD tool, but which confers intermediate resistance.^18^ The latter can be reconfigured in future versions.

There are several alternative analysis tools that predict resistance using MRSA genomes, the majority of which are used by researchers and require considerable informatics expertise. Nullarbor (https://github.com/tseemann/nullarbor) is a command line-based pipeline that requires basic computational expertise for its installation and use. The Center for Genomic Epidemiology in Denmark has developed an open-access web-based tool for resistance prediction,^19^ which utilizes the ResFinder database, but the output requires further informatics processing to infer resistance for individual drugs. Pathogenwatch (https://pathogen.watch) and the bioMérieux EpiSeq^TM^ system (https://www.biomerieux-episeq.com/) are the most comparable to the NGD tool. Pathogenwatch is an open-access tool developed by the Centre for Genomic Surveillance, which can predict drug resistance in an automated manner and has recently allowed upload of the raw FASTQ files. However, the concordance of this tool with phenotype has not yet been determined. The bioMérieux EpiSeq^TM^ system also requires data files (FASTQ or assembled FASTA) to be uploaded, which are then analysed in a cloud service. Fee-for-service analysis includes fully automated resistome characterization. EpiSeq^TM^ has been evaluated to investigate an increased incidence of *S. aureus* bloodstream infections in a neonatal ICU in France.^20^ In addition, machine-learning approaches such as AdaBoost have been developed that support upload of the assembled genome or sequence reads to detect antimicrobial resistance-conferring genes.^21^ The platform can be used to detect genes encoding resistance for a number of bacterial species including *S. aureus*. Since there are multiple online tools and databases of genetic determinants of resistance for *S. aureus*, a comparison of the predictive performance of the NGD tool with these tools will be important.

### Conclusions

The provision of rapid, accurate tools that can predict phenotypic drug susceptibility and output data in a format that can be readily used and interpreted by staff provides the opportunity to evaluate the impact of this for antibiotic stewardship and patient care, and determine whether these tools could be implemented into routine clinical care.

## Supplementary Material

dkz570_Supplementary_Data

## References

[1] Peacock SJ, Parkhill J, Brown NM. Changing the paradigm for hospital outbreak detection by leading with genomic surveillance of nosocomial pathogens. Microbiology 2018; 164: 1213–9. 30052172 10.1099/mic.0.000700 PMC7611365

[2] Harris SR, Cartwright EJ, Torok ME et al Whole-genome sequencing for analysis of an outbreak of meticillin-resistant *Staphylococcus aureus*: a descriptive study. Lancet Infect Dis 2013; 13: 130–6. 23158674 10.1016/S1473-3099(12)70268-2 PMC3556525

[3] Torok ME, Harris SR, Cartwright EJ et al Zero tolerance for healthcare-associated MRSA bacteraemia: is it realistic? J Antimicrob Chemother 2014; 69: 2238–45. 24788657 10.1093/jac/dku128 PMC4100711

[4] Coll F, Harrison EM, Toleman MS et al Longitudinal genomic surveillance of MRSA in the UK reveals transmission patterns in hospitals and the community. Sci Transl Med 2017; 9: eaak9745. 10.1126/scitranslmed.aak9745 PMC5683347 29070701

[5] Tosas Auguet O, Stabler RA, Betley J et al Frequent undetected ward-based methicillin-resistant *Staphylococcus aureus* transmission linked to patient sharing between hospitals. Clin Infect Dis 2018; 66: 840–8. 29095965 10.1093/cid/cix901 PMC5850096

[6] Gordon NC, Pichon B, Golubchik T et al Whole-genome sequencing reveals the contribution of long-term carriers in *Staphylococcus aureus* outbreak investigation. J Clin Microbiol 2017; 55: 2188–97. 28468851 10.1128/JCM.00363-17 PMC5483921

[7] The CRyPTIC Consortium and the 100,000 Genomes Project, Allix-Beguec C, Arandjelovic I et al Prediction of susceptibility to first-line tuberculosis drugs by DNA sequencing. N Engl J Med 2018; 379: 1403–15. 30280646 10.1056/NEJMoa1800474 PMC6121966

[8] Satta G, Lipman M, Smith GP et al *Mycobacterium tuberculosis* and whole-genome sequencing: how close are we to unleashing its full potential? Clin Microbiol Infect 2018; 24: 604–9. 29108952 10.1016/j.cmi.2017.10.030

[9] Baker S, Thomson N, Weill FX et al Genomic insights into the emergence and spread of antimicrobial-resistant bacterial pathogens. Science 2018; 360: 733–8. 29773743 10.1126/science.aar3777 PMC6510332

[10] Salipante SJ, SenGupta DJ, Cummings LA et al Application of whole-genome sequencing for bacterial strain typing in molecular epidemiology. J Clin Microbiol 2015; 53: 1072–9. 25631811 10.1128/JCM.03385-14 PMC4365209

[11] Blane B, Raven KE, Leek D et al Rapid sequencing of MRSA direct from clinical plates in a routine microbiology laboratory. J Antimicrob Chemother 2019; 74: 2153–6. 31039248 10.1093/jac/dkz170 PMC6640301

[12] Gordon NC, Price JR, Cole K et al Prediction of *Staphylococcus aureus* antimicrobial resistance by whole-genome sequencing. J Clin Microbiol 2014; 52: 1182–91. 24501024 10.1128/JCM.03117-13 PMC3993491

[13] Aanensen DM, Feil EJ, Holden MT et al Whole-genome sequencing for routine pathogen surveillance in public health: a population snapshot of invasive *Staphylococcus aureus* in Europe. MBio 2016; 7: e00444-16. 10.1128/mBio.00444-16 PMC4959656 27150362

[14] Bolger AM, Lohse M, Usadel B. Trimmomatic: a flexible trimmer for Illumina sequence data. Bioinformatics 2014; 30: 2114–20. 24695404 10.1093/bioinformatics/btu170 PMC4103590

[15] Haaber J, Penades JR, Ingmer H. Transfer of antibiotic resistance in *Staphylococcus aureus*. Trends Microbiol 2017; 25: 893–905. 28641931 10.1016/j.tim.2017.05.011

[16] Castanheira M, Watters AA, Bell JM et al Fusidic acid resistance rates and prevalence of resistance mechanisms among *Staphylococcus* spp. isolated in North America and Australia, 2007-2008. Antimicrob Agents Chemother 2010; 54: 3614–7. 20566766 10.1128/AAC.01390-09 PMC2934946

[17] Mason A, Foster D, Bradley P et al Accuracy of different bioinformatics methods in detecting antibiotic resistance and virulence factors from *Staphylococcus aureus* whole-genome sequences. J Clin Microbiol 2018; 56: e01815-17. 29925638 10.1128/JCM.01815-17 PMC6113501

[18] Lee AS, Gizard Y, Empel J et al Mupirocin-induced mutations in *ileS* in various genetic backgrounds of methicillin-resistant *Staphylococcus aureus*. J Clin Microbiol 2014; 52: 3749–54. 25122856 10.1128/JCM.01010-14 PMC4187773

[19] Thomsen MC, Ahrenfeldt J, Cisneros JL et al A bacterial analysis platform: an integrated system for analysing bacterial whole genome sequencing data for clinical diagnostics and surveillance. PLoS One 2016; 11: e0157718. 27327771 10.1371/journal.pone.0157718 PMC4915688

[20] Rouard C, Bourgeois-Nicolaos N, Rahajamanana L et al Evaluation of an ‘all-in-one’ seven-day whole-genome sequencing solution in the investigation of a *Staphylococcus aureus* outbreak in a neonatal intensive care unit. J Hosp Infect 2019; 102: 297–303. 30771369 10.1016/j.jhin.2019.01.029

[21] Davis JJ, Boisvert S, Brettin T et al Antimicrobial resistance prediction in PATRIC and RAST. Sci Rep 2016; 6: 27930. 27297683 10.1038/srep27930 PMC4906388

