# Supplementary material for: Evaluation of a fully automated bioinformatics tool to predict antibiotic resistance from MRSA genomes
Source: J Antimicrob Chemother. 2020 Feb 5;75(5):1117–22. doi: 10.1093/jac/dkz570 (PMC7177496; doi:10.1093/jac/dkz570)
Supplement: dkz570_Supplementary_Data [file dkz570_supplementary_data.zip › SupplementaryFigs1726.doc]

**Supplementary data**

**Supplementary Figures to: Evaluation of a fully automated bioinformatics tool to predict antibiotic resistance from MRSA genomes**

Narender Kumar, Kathy E. Raven, Beth Blane, Danielle Leek, Nicholas M. Brown, Eugene Bragin, Paul A. Rhodes, Julian Parkhill, Sharon J. Peacock

**Figure S1.** Distribution of multilocus sequence types for the 778 study MRSA isolates. Individual STs are shown for those containing at least 7 isolates, with the remainder placed into a single category terms “Others”.

**Figure S2.** Phenotypic susceptibility testing results based on disc diffusion assay performed by the routine laboratory for the 778 study MRSA isolates. The number shown above each column represents the total number of isolates tested again each drug. Antibiotic names are abbreviated as CEF: cefoxitin; ERY: erythromycin; TET: tetracycline; RIF: rifampicin; FUS: fusidic acid; GEN: gentamicin; CHL: chloramphenicol; MUP: mupirocin; LIN: linezolid; CIP: ciprofloxacin; TRI: trimethoprim.

**Figure S3.** The antibiotics involved in the 134 discrepant results between phenotypic susceptibility results and genetic prediction of susceptible/resistant. The number shown above each column represents the number of discrepant isolates per drug. Antibiotic manes are abbreviated as CEF: cefoxitin; ERY: erythromycin; TET: tetracycline; RIF: rifampicin; FUS: fusidic acid; GEN: gentamicin; CHL: chloramphenicol; MUP: mupirocin; LIN: linezolid; CIP: ciprofloxacin; TRI: trimethoprim.
